# Sex‐specific effects of maternal weight loss on offspring cardiometabolic outcomes in the obese preeclamptic‐like mouse model, BPH/5

**DOI:** 10.14814/phy2.15444

**Published:** 2022-09-06

**Authors:** Kalie F. Beckers, Christopher J. Schulz, Juliet P. Flanagan, Daniella M. Adams, Viviane C.L. Gomes, Chin‐Chi Liu, Gary W. Childers, Jenny L. Sones

**Affiliations:** ^1^ Veterinary Clinical Sciences, School of Veterinary Medicine Louisiana State University Baton Rouge Louisiana USA; ^2^ Department of Biological Sciences Southeastern Louisiana University Hammond Louisiana USA; ^3^ Pennington Biomedical Research Center Louisiana State University Baton Rouge Louisiana USA

**Keywords:** hypertensionh, obesity, preeclampsia, sex as a biological variable

## Abstract

AbstractPreeclampsia (PE) is a hypertensive disorder that impacts 2–8% of pregnant women worldwide. It is characterized by new onset hypertension during the second half of gestation and is a leading cause of maternal and fetal morbidity/mortality. Maternal obesity increases the risk of PE and is a key predictor of childhood obesity and potentially offspring cardiometabolic complications in a sex‐dependent manner. The influence of the maternal obesogenic environment, with superimposed PE, on offspring development into adulthood is unknown. Obese BPH/5 mice spontaneously exhibit late‐gestational hypertension, fetal demise and growth restriction, and excessive gestational weight gain. BPH/5 females have improved pregnancy outcomes when maternal weight loss via pair‐feeding is imposed beginning at conception. We hypothesized that phenotypic differences between female and male BPH/5 offspring can be influenced by pair feeding BPH/5 dams during pregnancy. BPH/5 pair‐fed dams have improved litter sizes and increased fetal body weights. BPH/5 offspring born to ad libitum dams have similar sex ratios, body weights, and fecal microbiome as well as increased blood pressure that is reduced in the dam pair‐fed offspring. Both BPH/5 male and female offspring born to pair‐fed dams have a reduction in adiposity and an altered gut microbiome, while only female offspring born to pair‐fed dams have decreased circulating leptin and white adipose tissue inflammatory cytokines. These sexually dimorphic results suggest that reduction in the maternal obesogenic environment in early pregnancy may play a greater role in female BPH/5 sex‐dependent cardiometabolic outcomes than males. Reprograming females may mitigate the transgenerational progression of cardiometabolic disease.

## 1 INTRODUCTION

Preeclampsia (PE) is characterized by late gestational onset of hypertension (systolic ≥140 mmHg or diastolic ≥90 mmHg), proteinuria, renal insufficiency, thrombocytopenia, hepatic dysfunction, and pulmonary edema (Leeman & Fontaine, 2008). PE affects up to 2–8% of pregnancies worldwide, making it a leading cause of maternal and fetal morbidity and mortality (Jeyabalan, 2013). The only known treatment is delivery of the fetus and the placenta, which can have deleterious consequences on the mother and offspring. PE may lead to significant offspring co‐morbidities, including small‐for‐gestational‐age (SGA) neonates due to fetal growth restriction (FGR) and pre‐term delivery‐associated sepsis, and could ultimately lead to cardiovascular and metabolic disease later in life (Jeyabalan, 2013). Obese women are 6 times more likely to develop hypertension than their lean counterparts (Stang & Huffman, 2016), and twice as likely to develop PE (Flegal et al., 2012). Maternal obesity also increases the risk of pregnancy complications including gestational diabetes mellitus, preterm delivery, Caesarian section, SGA and stillbirths (Lim & Mahmood, 2015; Lutsiv et al., 2015; MacInnis et al., 2016; Mission et al., 2015; Sohlberg et al., 2012; Stang & Huffman, 2016; Yao et al., 2017). In addition, maternal obesity predisposes offspring to cardiovascular, metabolic, and neurological disorders in adulthood (LaCoursiere et al., 2010; Nguyen et al., 2015; Rivera et al., 2015; Stang & Huffman, 2016). This emphasizes the need for therapeutic interventions for obese mothers and their offspring. Studying the effects on the offspring in a sex dependent manner is equally as important. In the United States, 37.9% of men and 41.1% of women are considered obese. According to WHO, while one in five women are diagnosed with hypertension, men have a one in four chance of developing the condition, making hypertension more prevalent and potentially a greater health risk in men (Gillis & Sullivan, 2016). Therefore, an effect of sex is hypothesized in the development of these conditions.

The BPH/5 female mouse spontaneously develops obesity and hypertension pre‐conception (Sutton et al., 2017), and superimposed PE when challenged with pregnancy (Davisson et al., 2002). The maternal obesogenic environment may play a role in offspring post‐natal development and metabolism. However, the future developmental and transgenerational impacts of male and female offspring gestating in obese mothers with superimposed PE is not completely understood. Previous research demonstrates that BPH5 offspring have sex‐dependent phenotypic differences. The BPH/5 female offspring present excessive catch up growth after birth, hyperphagia, obesity, cardiomegaly, increased blood pressure, and hyperleptinemia with leptin resistance (Sutton et al., 2017). However, the adult male BPH/5 offspring are not hyperphagic and are not obese, but do exhibit increased white adipose tissue mass and cardiomegaly with left ventricular hypertrophy (Beckers et al., 2021).

Herein, we propose the BPH/5 as a model for studying offspring born to PE dams in a sex‐dependent manner. A pair‐feeding paradigm was used to match the food intake of the pregnant BPH/5 mice to the normal intake of the lean C57 controls of similar age and day of gestation. The BPH/5 offspring outcomes were then investigated after attenuation of maternal obesity during pregnancy. The metabolic imbalances and cardiac risk were investigated in the offspring of obese, ad libitum‐fed, BPH/5 dams and compared to the offspring of lean, pair‐fed, BPH/5 dams. Previous research has shown the benefits of pair‐feeding dams, by decreasing adult body weight and reducing adiposity in BPH/5 female offspring (Sutton et al., 2017). We hypothesized that cardiometabolic risk differences exist between female and male BPH/5 offspring and that pair feeding BPH/5 dams during pregnancy would reprogram offspring to improve these outcomes.

## 2 METHODS

### 2.1 Animal experiments

Adult (2–6 months of age) female and male BPH/5 mice were used in this study. BPH5 mice were a gift from Dr. Robin Davisson, Cornell University and maintained as an in house colony at Louisiana State University. Mice were housed in standard cages placed in a temperature and humidity‐controlled facility, maintained on a 12‐h light/dark cycle, and fed standard mouse chow (Purina 5001) with water available ad libitum. Two groups of dams were used: (1) Ad libitum BPH/5 (AL) (*n* > 6) and (2) Pair‐fed BPH/5 (PF) (*n* = 6). Group 2 was randomly selected BPH/5 dams that were pair‐fed to mirror C57 food intake beginning at detection of copulatory plug, as previously described (Reijnders et al., 2019). C57 mice have been used as control mice in previous BPH/5 studies, as they were used in the original eight‐way cross to derive the BPH/5 strain (Davisson et al., 2002; Reijnders et al., 2019; Sones et al., 2016). Intrastrain timed matings were performed, and detection of a copulatory plug was designated as embryonic day 0.5 (e0.5). Two males and two females were randomly selected per litter across at least two litters of AL and PF dams. After parturition, these offspring were allowed to mature to adulthood, eating ad libitum. Four offspring groups were studied: (a) BPH/5 female offspring born to ad libitum fed dams: AL/AL^F^, (b) BPH/5 male offspring born to ad libitum dams: AL/AL^M^, (c) BPH/5 female offspring born to pair‐fed dams: PF/AL^F^, and (d) BPH/5 male offspring born to pair‐fed dams: PF/AL^M^. All animal studies were approved by the Louisiana State University School of Veterinary Medicine and Pennington Biomedical Research Center IACUC committees.

### 2.2 Pair‐feeding dam's protocol

A cohort of pregnant BPH/5 female (*n* = 6) mice were restricted to 3 g of Purina rodent normal chow per day. Normal chow food intake of ad libitum‐fed 6‐week‐old nonpregnant C57 and BPH/5 mice was measured concurrently for 14 days. The pair‐fed cohort of BPH/5 mice received food intake matched to C57BL/6 counterparts (~25% less calories than their ad libitum‐fed BPH/5 littermates during that time) (Reijnders et al., 2019).

### 2.3 Samples collected

Litter size, fetal and postnatal three‐week‐old body weights, adult body weights, and weights of liver, hearts, inguinal subcutaneous white adipose tissue (WAT), and visceral WAT from the peri‐renal depot and the reproductive depot of both AL and PF groups of adult male and female BPH/5 age‐matched (8–10 weeks) offspring. Litter size was used as inclusion criteria. Namely, body weights were measured from pups born to BPH/5 with litters sizes greater than or equal to 4 pups as PF BPH/5 dams did not have litters with less than 4 pups. Fetal livers weighed and expressed as a ratio to body weight to rule out asymmetric growth (Swanson & David, 2015). Offspring were fed an ad libitum normal chow diet until phenotypic analysis at adulthood. Additionally, fresh fecal samples were collected in all offspring groups between 8–12 weeks of age.

### 2.4 PCR of SRY from genomic

Tail snips (1.2 cm) were collected post‐mortem from BPH/5 (*n* = 26) gestational day 18.5 (e18.5) pups. The tails were cut into smaller pieces and DNA was extracted using Qiagen DNeasy Blood & Tissue Kit according to manufacturer's (Qiagen) protocols. DNA quantity and quality was assessed by spectrophotometry (Thermofisher Scientific, Nanodrop 200). To determine the sex of each pup, a final total reaction of 25 μl PCR was performed using 0.2 ul of each SRY (F‐AACAACTGGGCTTTGCACATT, R‐GTTTATCAGGTTTCTCTCTAGC) primer, 12.5 μl of Taq, 8.5 μl of RNAse‐free H2O, and 2 μl of DNA. The PCR thermal conditions were adapted from a standard PCR genotyping protocol from the Mouse Metabolic Phenotyping Centers Protocols (https://mmpc.org/shared/document.aspx?id=260&doctype=Protocol). The cycle was as followed: 94°C for 5 min, 10 cycles of 94°C for 15 s, 65°C for 30s, 72°C for 40s, 30 cycles of 94°C for 15 s, 55°C for 30s, 72°C for 40s, 72°C for 15 min, and hold at 15°C. A 2% agarose gel was used to identified SRY positive bands with two male and female adult controls.

### 2.5 Leptin ELISA

Blood was collected via cardiac puncture, allowed to clot at room temperature for 90 min, centrifuged at 3500 rpm for 20 min, and then stored at −80°C until it was assayed. A commercially available leptin ELISA was performed according to manufacturer's instructions (Cayman Chemicals) with serum as previously described (Sutton et al., 2017). The sensitivity of the assay was 50 pg/ml.

### 2.6 Quantitative reverse‐transcription PCR of WAT

Total RNA was extracted from female and male visceral WAT and subcutaneous WAT using TRIzol according to manufacturer's (Qiagen) guidelines to examine *TNFa* (F‐GAACTGGCAGAAGAGGCACT, R‐AGGGTCTGGGCCATAGAACT (Sutton et al., 2017)), *IL‐6* (F‐TGGCTAAGGACCAAGACCATCCAA, R‐AACGCACTAGGTTTGCCGAGTAGA (Sutton et al.,  2017)), and *Ptgs‐2* (F‐ACTGGGCCATGGAGTGGACTTAAA, R‐AACTGCAGGTTCTCAGGGATGTGA (Sones et al., 2016)) expression levels. RNA quality and quantity were assessed by spectrophotometry (NanoDrop). One thousand nanograms was used for reverse transcription using the qScript cDNA kit (Quanta BioSciences). Each qPCR was performed in triplicate with an ABI 7500 Fast Thermocycler (Applied Bioscience) using SYBR Green (QuantaBioSciences) using 25 ng cDNA. Data were analyzed using the ΔΔCT method, and results were normalized to 18 s gene (Sones et al., 2014; Sones et al., 2016).

### 2.7 Radiotelemetric measurement of blood pressure and heart rate

Pair‐fed dams, (*n* = 4) and adult offspring born to pair‐fed dams (*n* = 4) and ad libitum dams (*n* = 13) underwent carotid implantation of telemetry (Data Sciences International, Saint Paul, MN) according to published methods (Davisson et al., 2002). Briefly, mice were anesthetized with isoflurane through inhalation for placement of a telemeter in the left carotid artery and transmitter body in the subcutaneous space. Mice were given carprofen (5 mg/kg subcutaneous) every 24 h for two days post‐surgery. Mice were allowed to recover for 7 days, followed by 4 days of heart rate (beats per min) and mean arterial pressure (MAP) recording in adult offspring mice and during the length of gestation in BPH/5 females mice as described (Davisson et al., 2002). Heart rate and MAP were interpreted by an observer blinded to the study design.

### 2.8 DNA sequencing

Microbial DNA was extracted from fecal samples using the Qiagen DNeasy PowerSoil extraction kits (Qiagen) according to manufacturer's protocol. The V4 variable region of the 16S rRNA gene was amplified with PCR primers 515/806 in a 30 cycle PCR using the DreamTaq Hot Start PCR Master Mix Kit (Thermoscieniftic). PCR was performed in 20 μl vol and included: 2 μl (7.5 μM concn) of forward and reverse primers, 12.5 μl of Hot Start Taq 2X Master Mix (New England BioLabs Inc.), 3.5 μl of deionized water, and 2 μl of sample DNA. Thermal cycle conditions were 95°C for 3 min for initial denaturing step, followed by 30 cycles of 95°C for 30 s, 50°C for 1 min, and 72°C for 1 min. PCR products were checked on a 2% agarose gel for correct product size formation (approx 350 bp). Michigan State University Genomics Core performed library preparation prior to Illumina MiSeq sequencing following the manufacturer's guidelines. Reagent controls using certified DNAfree water were run through library preparation and PCR and did not generate libraries. For quality control, samples submitted for sequencing included a random blank sample of technical replicates.

### 2.9 Bioinformatics

Initial quality screening, demultiplexing, amplicon sequence variant (ASV) inference and chimera removal were performed using the DADA2 package. ASVs were classified using Greengenes v13.8 database (DeSantis et al., 2006). For contamination of libraries, background sequences were removed using Decontam package. DESeq2 will be used to correct for different library read depths and to detect ASVs of differing abundances between treatment groups. Microbial Community analysis (Alpha and Beta diversity) was performed using vegan R package.

### 2.10 Statistical analysis

Statistical analysis was performed using GraphPad Prism version 9. Two‐way ANOVA with Tukey's post hoc test and/or a Student's *t*‐test were used. Normality of residuals from the models were assessed and confirmed via Shapiro–Wilk tests and quantile‐quantile (Q‐Q) plots. All figures were presented as means± SEM. *p* values <0.05 were considered significant.

## 3 RESULTS

### 3.1 Sex differences in offspring born to obese hypertensive BPH/5 dams is not apparent until adulthood

**FIGURE 1:**
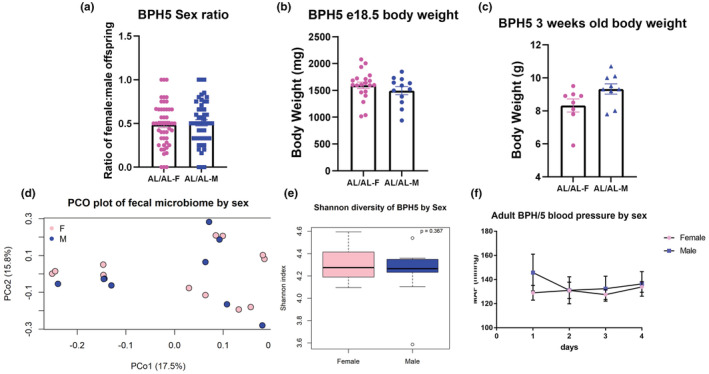
No differences were found between sexes in BPH5 offspring in sex ratio, fetal body weight, body weight after lactational growth, fecal microbiome, or blood pressure demonstrating that BPH5 is a good model to study offspring born to PE dams in a sex‐dependent manner. (a) Sex ratio was measured in embryonic day (e18.5) in BPH5 AL/AL offspring, no difference was found (*n* = 49). (b) Body weights were measured in e18.5 day fetuses using SRY to identify sex, no difference was found (*n* = 12–20). (c) Body weights were measured in prepubertal BPH5 AL/AL offspring similar weight was demonstrated in both males and females (*n* = 8–32). (d) Microbial community composition of fecal samples was investigated using PERMANOVA with Bray–Curtis dissimilarity of 16S Sequences variants detected (ASVs) relative abundance, communities were found to be not significantly different (*n* = 9–12). (e) Shannon diversity, measuring alpha diversity, showed there was no difference in male and female fecal communities. (*n* = 9–12). (f) Blood pressure was measured using radiotelemetry devices over 4 days. Although BPH5 AL/AL male and female both had increased mean arterial pressure, it was not significantly different between sexes (*n* = 5–8). *p* > 0.05

To study BPH/5 offspring outcomes in a sex‐dependent manner mechanistically, including sex distribution and morphometrics were characterized. We have previously published that BPH/5 have symmetrical FGR (Davisson et al., 2002; Dokras et al., 2006; Sones et al., 2016), but for the first time we assessed this by sex. The offspring sex ratio (50.7% males and 48.8% females within a litter) observed no significant difference between BPH5 male and female offspring born to ad libitum dams (Figure 1a). BPH/5 e18.5 fetuses were genotyped for expression of the SRY gene, indicative of male sex (Hacker et al., 1995). No body weight differences were observed between BPH/5 males and females at e18.5 (Figure 1b) or at weaning in prepubertal (~3 weeks of age) mice (Figure 1c). Thus, lactation had no effect on body weight between male and females, whereas a difference was not observed in adulthood (8 weeks of age), with comparable body weight between the BPH/5 males and females (Table S1), which have increased body weight compared to C57 age‐matched female mice (Sutton et al., 2017).

Microbial community composition of fecal samples was investigated using PERMANOVA with Bray–Curtis dissimilarity of 16S Sequences variants detected (ASVs) relative abundance. Shannon diversity, measuring alpha diversity, showed there was no difference in male and female fecal communities. The community composition of BPH/5 ad libitum male and female offspring were not significantly different using a PERMANOVA to analyze Bray–Curtis dissimilarity and alpha diversity using Shannon diversity matrices (Figure 1d,e). Likewise, no difference was found between sexes in the BPH/5 ad libitum offspring mean arterial pressure (Figure 1f).

### 3.2 Improved BPH/5 maternal and fetal pregnancy outcomes with pair‐feeding

**FIGURE 2:**
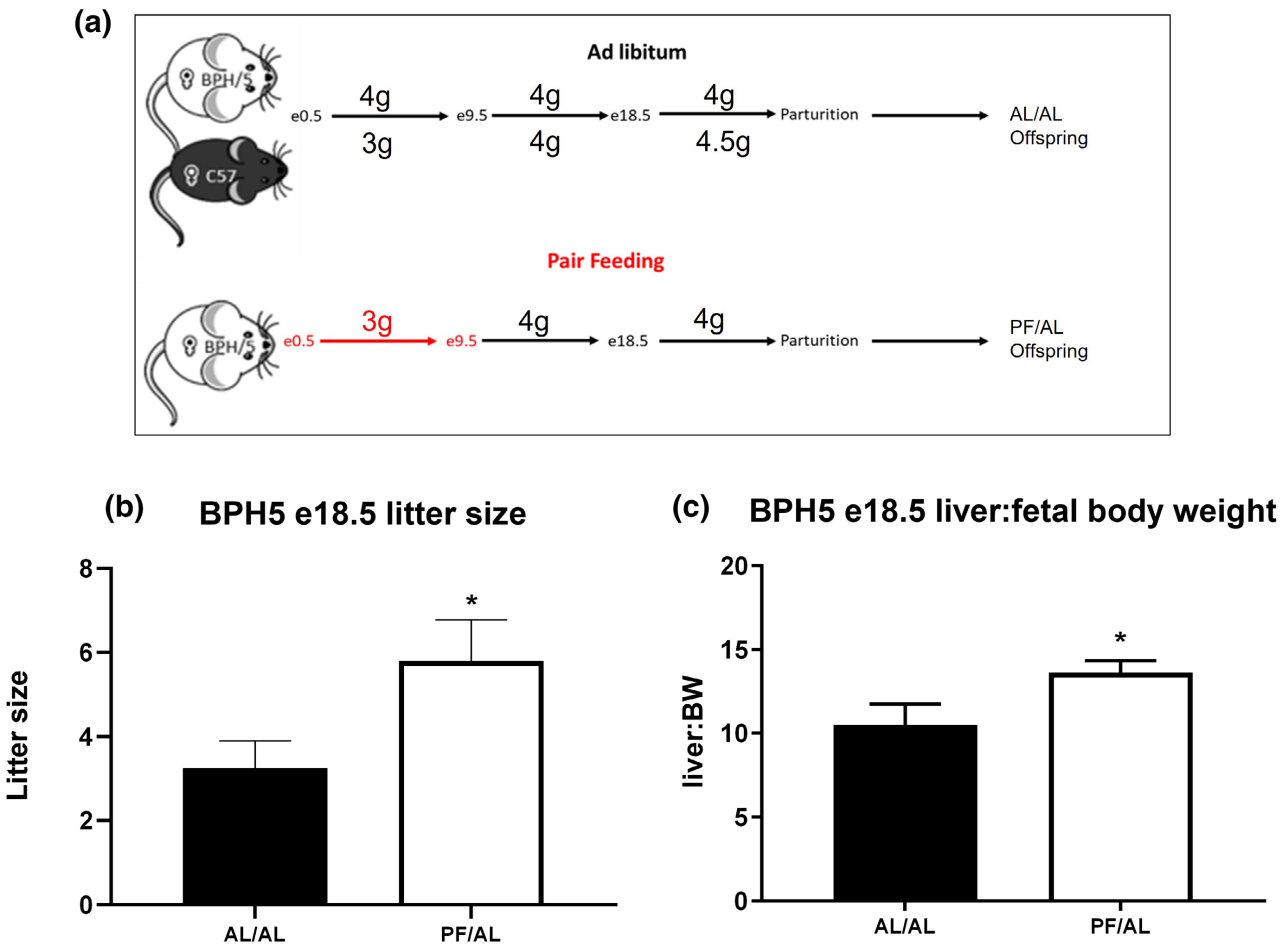
Pair feeding BPH/5 female mice beginning at conception normalizes litter size and fetal weight by e18.5. (a) Depiction of the pair‐feeding paradigm used in this study with the BPH5 pair‐fed group matching the food intake of control C57 strain (g = grams). (b) Litter size of BPH/5 ad libitum and pair‐fed was measured at embryonic day (e)18.5, showing an increase in number of pups born to pair‐fed dams. (*n* = 4). (c) In utero fetal symmetrical growth was measured by expressing liver weight to body weight as a ratio at day e18.5 (*n* = 3–9). *p* < 0.05.

After introduction of the pair‐feeding intervention to BPH/5 dams (PF/AL), e18.5 pregnancy outcomes were assessed and compared to ad libitum fed BPH/5 (AL/AL) (Figure 2a). The pair‐feeding paradigm was used to match the calorie intake of the obese BPH/5 to the lean C57 control during pregnancy. Previous literature shown that BPH5 are hyperphagic during early pregnancy, thus the pair‐feeding intervention only imposes caloric restriction for this first 9 days of pregnancy (Reijnders et al., 2019). Gestational day 9.5 represents similarity to the first trimester in women and during placenta formation (Sones & Davisson, 2016). We have previously published that BPH/5 have evidence of in utero fetal demise with decreased litter sizes at mid‐gestation due to resorptions (Davisson et al., 2002; Sones & Davisson, 2016). BPH/5 litter size was significantly higher in BPH/5 PF/AL (5.8 ± 0.97) versus BPH/5 AL/AL (mean = 3.2 ± 0.65) (Figure 2b). Liver to body weight ratio was increased in offspring born to PF dams on e18.5 (Figure 2c). BPH/5 have previously shown to an increase in late gestational blood pressure from non‐pregnant baseline (Davisson et al., 2002; Sones & Davisson, 2016), when pair‐feeding was implemented we fail to see rise from baseline mean arterial pressure at any timepoint during pregnancy (Figure S1).

### 3.3 Cardiometabolic phenotypic differences by sex found in offspring born to BPH/5 dams after maternal weight loss

**FIGURE 3:**
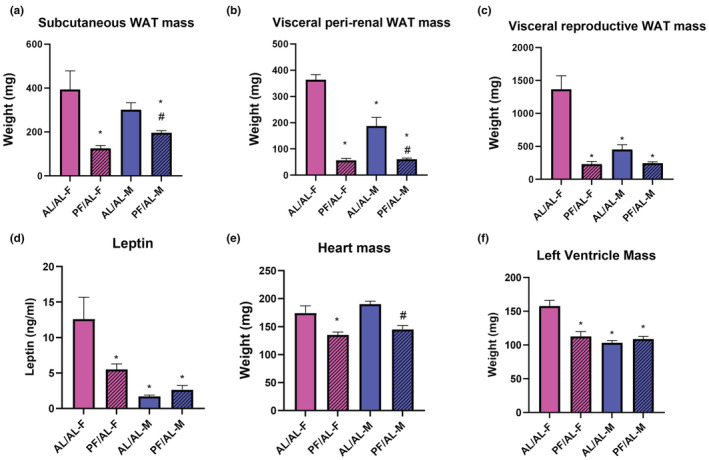
Pair‐feeding BPH/5 dams attenuates features of cardiometabolic disease risk in offspring in a sex‐dependent manner. (a) BPH5 offspring born to pair‐fed dams have significantly decreased subcutaneous WAT compared to AL/AL offspring. (*n* = 3–18/group). (b) BPH5 offspring born to pair‐fed dams have significantly decreased visceral peri‐renal WAT compared to AL/AL offspring, with AL/AL‐M significantly less than AL/AL‐F. (*n* = 3–18/group). (c) BPH5 offspring born to pair‐fed dams have significantly decreased visceral reproductive WAT compared to AL/AL offspring, with AL/AL‐M significantly less than AL/AL‐F. (*n* = 3–18/group). (d) Circulating leptin was measured and significantly reduced in PF/AL‐F, while it remained low in the BPH5 male offspring. (*n* = 5–8). (e) BPH5 offspring born to pair‐fed dams demonstrate a reduction in heart mass compared to AL/AL offspring of the same sex. (f) Left ventricular mass was measured and significantly reduced in PF/AL‐F, while it remained low in the BPH5 male offspring. (*n* = 3–8). (*p < 0.05 vs. AL/AL‐F, #*p* < 0.05 vs. AL/AL‐M)

When assessing pair fed BPH/5 outcomes by sex (female: PF/AL^F^; male: PF/AL^M^), adult body weight in the BPH/5 PF/AL^F^ and PF/AL^M^ demonstrated 15% and 17% reduction compared to BPH/5 AL/AL^F^ and AL/AL^M^, respectively (Table S1). BPH/5 adult ad libitum fed offspring (AL/AL^F^) have evidence of cardiometabolic disease with increased pro‐inflammatory visceral and subcutaneous WAT, hyperleptinemia, and heart enlargement with hypertension versus C57^F^ controls as previously published (Sutton et al., 2017). BPH/5 AL/AL^M^ have increased visceral and subcutaneous WAT mass versus C57 controls males (Beckers et al., 2021). Prevention of maternal obesity in the PF/AL^F^ and PF/AL^M^ offspring reduces WAT mass in the subcutaneous and visceral WAT depots, and the reproductive WAT is decreased only in BPH/5 PF/AL^F^ (Figure 3a‐c). BPH/5 PF/AL^F^ offspring had reduced serum leptin levels when compared to BPH/5 AL/AL^F^ offspring (Figure 3d). Cardiomegaly was also attenuated in PF/AL^F^ and PF/AL^M^ offspring (Figure 3e). BPH/5 AL/AL^F^ offspring were found to have significant left ventricular hypertrophy that was attenuated in PF/AL^F^ (Figure 3f).

**FIGURE 4:**
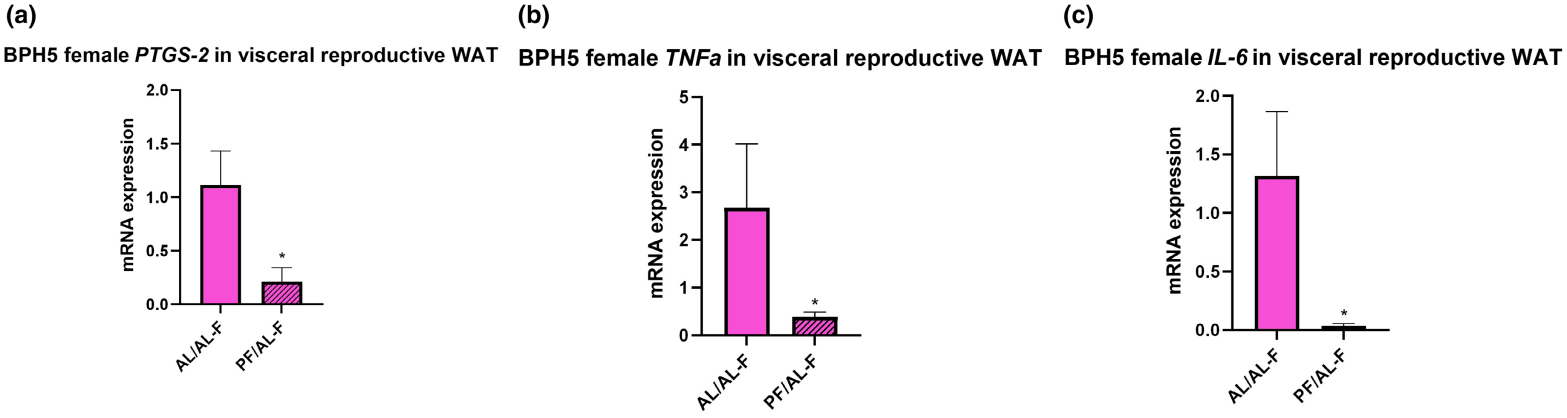
Pair‐feeding BPH/5 dams decreases pro‐inflammatory cytokine expression in visceral reproductive white adipose tissue in female offspring. BPH5 AL/AL‐F offspring exhibit increased inflammatory mediators within visceral reproductive white adipose tissue (WAT). (a) Using real‐time PCR, BPH5 PF/AL have significantly decreased prostaglandin synthase 2 (Ptgs‐2) relative mRNA expression compared to AL/AL‐F; (b) Tumor necrosis factor (TNFa) relative mRNA expression was significantly decreased compared to AL/AL‐F; and (c) interleukin‐6 (IL‐6) mRNA expression was significantly decreased compared to AL/AL‐F. *n* = 3–8, *p < 0.05 when compared to AL/AL‐F

As previously shown, BPH/5 AL/AL^F^ offspring have an upregulation in visceral reproductive WAT *IL‐6, PTGS‐2*, and *TNFa* expression (Sutton et al., 2017). BPH/5 PF/AL^F^ offspring demonstrated decreased *IL‐6, PTGS‐2*, and *TNFa* expression in visceral reproductive WAT (Figure 4a‐c). While BPH/5 AL/AL^M^ showed an increase in visceral peri‐renal *TNFa* and subcutaneous WAT *Ptgs‐2* and *IL‐6* expression (Beckers et al., 2021), no change was found in PF/AL^M^ offspring (data not shown).

**FIGURE 5:**
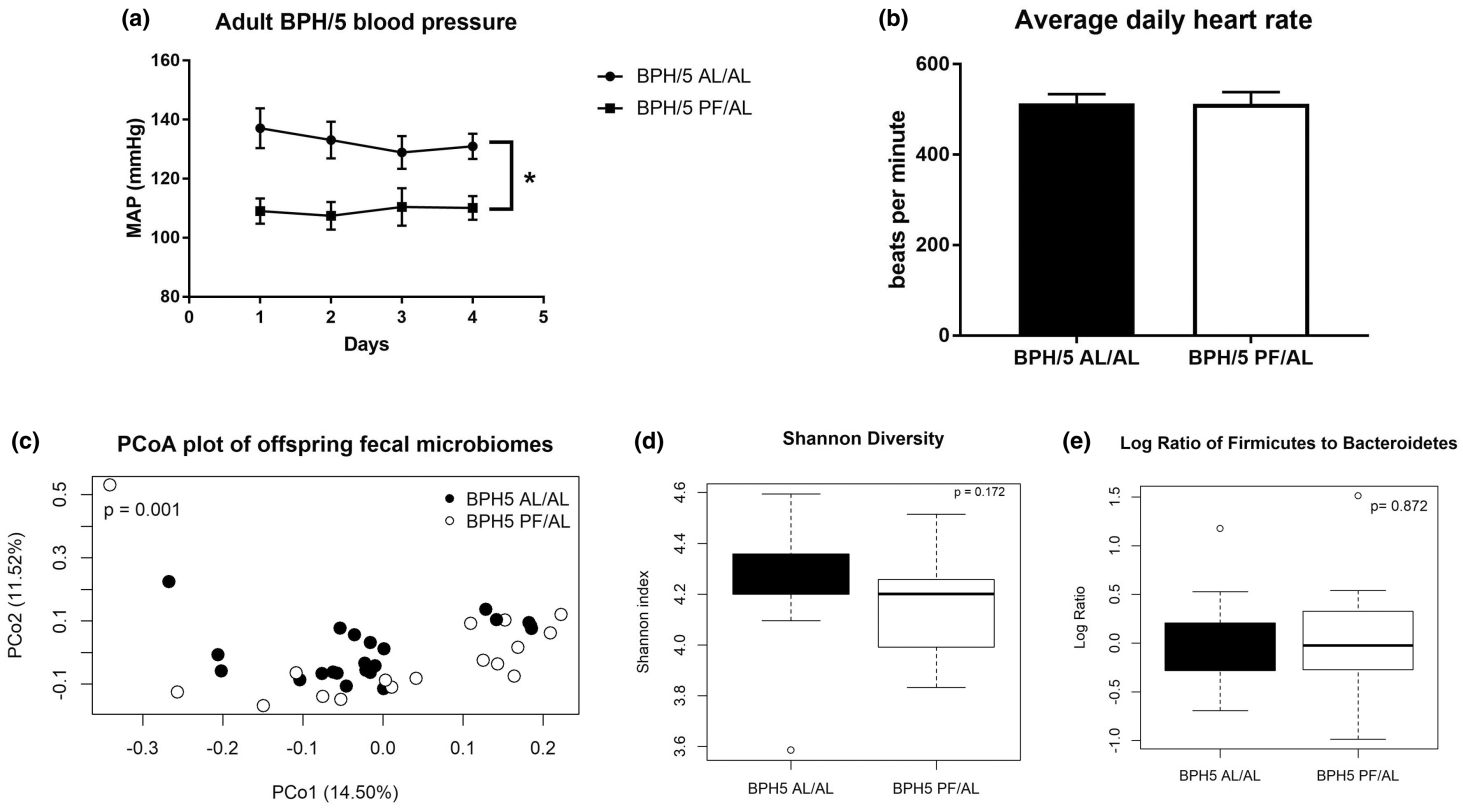
Pair‐feeding BPH/5 dams improve hypertension and alter the fecal microbiome in offspring. (a) BPH5 PF/AL offspring demonstrate decreased mean arterial pressure compared to AL/AL offspring as measured over 4 consecutive days (*n* = 4–13, *p* < 0.05). (b) Daily average heart rates measured as beats per min were not different (*n* = 4–13, *p* > 0.05). (c) BPH5 PF/AL offspring microbial community composition of fecal samples was investigated using PERMANOVA with Bray–Curtis dissimilarity of 16S Sequences variants detected (ASVs) relative abundance and the communities were found to be significantly different when compared to AL/AL offspring (*n* = 9–12, *p* = 0.001). (d) Shannon diversity, measuring alpha diversity, showed there was no difference in PF/AL offspring and AL/AL in fecal samples (*n* = 9–12, *p* > 0.05). (e) The log ratio of Firmicutes to Bacteroidetes, a known indicator of obesity, was not significantly different between PF/AL and AL/AL. (*n* = 9–12, *p* > 0.05)

Both BPH5 AL/AL^F^ and AL/AL^M^ offspring have MAP greater than 100 mmHg (Figure 1f), which is reduced in the BPH/5 PF/AL^F^ and PF/AL^M^ offspring (Figure 5a, *p* < 0.05). While blood pressure is decreased, the heart rate was not different (Figure 5b, *p* > 0.05). Beta diversity analysis of offspring fecal microbial community samples, using a PERMANOVA with Bray–Curtis dissimilarity comparing BPH5 AL/AL and PF/AL offspring, was significantly different (Figure 5c, *p* = 0.001). No difference was found between groups using Shannon diversity examining alpha diversity or between the log ratio of Firmicutes to Bacteroidetes (Figure 5d‐e, *p* > 0.05).

## 4 DISCUSSION

Sex as a biological variable is an important consideration when testing physiological hypotheses. Herein we describe the sex differences in female and male BPH/5 mice. The BPH/5 mouse has been utilized for over 20 years as a model of superimposed PE; however, offspring differences in a sex‐dependent manner have not been directly compared. Utilizing spontaneous animal models of cardiometabolic disease allows for exploration of these genetic contributors to hypertension in a sex‐dependent manner. BPH/5 females have pre‐existing cardiometabolic risk before pregnancy (hyperphagia, obesity and leptin resistance, and hypoestrogenemia) (Sutton et al., 2017) and their phenotype is unique from BPH/5 males. Offspring of PE‐like BPH/5 dams have intrauterine FGR and smaller birthweights when compared to C57 aged‐matched counter‐parts (Davisson et al., 2002; Dokras et al., 2006; Sones et al., 2016). BPH/5 female ad libitum‐fed (AL/AL^F^) offspring exhibit accelerated catch up growth, i.e., small for gestational age at birth, then overweight by adulthood (Sutton et al., 2017). BPH/5 male ad libitum‐fed (AL/AL^M^) offspring have similar prepubertal and adult body weights when compared to C57 age‐matched controls (Beckers et al., 2021).

In this study, we first sought to determine the sex ratios of live BPH/5 offspring. There is an even number of BPH/5 female and male offspring born within a litter with similar bodyweights between both sexes at e18.5 and at weaning (3 weeks of age). Rodents, like most mammals, have a similar number of male to female offspring, although in the diseased state this ratio is altered which promotes healthier males and helps preserve the species (Rugh, n.d.; Koskela et al., 2009). Interestingly in the case of BPH/5, which has been studied extensively as a model of hypertension, the sex ratio at birth is equivalent and thus allows for equal analysis of female and male offspring outcomes amongst littermates. Then, we determined for the first time, using SRY genotyping, that male and female pups at e18.5 are equally impacted by symmetrical FGR in utero and this is maintained throughout the lactational period. Furthermore, by adulthood, BPH/5 female and male offspring have similar body weights, fecal microbiomes, and mean arterial pressure, which is in the hypertensive range. This direct comparison between BPH/5 female and male littermates confirms that BPH/5 females have evidence of adult onset obesity as previously reported (Sutton et al., 2017), as opposed to males. Birth weight is inversely related to blood pressure in men (Curhan, Willett, et al., 1996) and women (Curhan, Chertow, et al., 1996). Vos et al showed that cardiovascular risk is two‐fold greater in low birth weight (LBW) men relative to LBW females in young adulthood (Vos et al., 2006), indicating a sex difference in susceptibility to cardiovascular risk may originate in fetal life. As with BPH/5 females, adult‐onset obesity needs to be considered when predicting cardiometabolic disease risk in humans.

Infants with LBW exhibit accelerated catch up growth and a more central pattern of fat distribution, reduced lean mass, and increased adiposity (Singhal et al., 2003; Walker et al., 2002). BPH/5 females demonstrate accelerated catch up growth, while both BPH/5 females and males have increased adiposity (Beckers et al., 2021; Reijnders et al., 2019). Using our published feeding paradigm to promote maternal weight loss in BPH/5 pregnancy (Reijnders et al., 2019), we show that pair‐feeding BPH/5 dams result in attenuation of LBW and increased litter size. Improved pregnancy outcomes in offspring with pair‐feeding are continued into BPH/5 adulthood, PF/AL^F^ and PF/AL^M^ offspring have altered body weight at 8 weeks of age, where they show a significant reduction in body weight compared to the ad libitum offspring. The reduction in body weight may be attributed to significant loss of subcutaneous and visceral WAT in both male and female BPH/5 offspring. Further studies assessing BPH/5 offspring body composition are warranted. The difference in body weight may be attributed to differences in soft tissue density, but further research and advanced serial whole‐body imaging are needed to assess lean versus fat mass as BPH/5 age.

Our BPH/5 dam pair‐feeding paradigm suggests in utero fetal reprogramming of morphometrics to subsequently improve offspring outcomes and prevent obesity into adulthood. Despite similar gestational and postnatal environments, BPH/5 males are not obese nor hyperphagic and do not exhibit hyperleptinemia as females do (Beckers et al., 2021). However, both BPH/5 females and males have heart enlargement, and increased blood pressure (Beckers et al., 2021; Sutton et al., 2017). Improving the BPH/5 maternal obesogenic environment by pair‐feeding the dams, results in reduction of WAT depot mass and heart enlargement in both male and female adult offspring, whilst lowering circulating leptin and left ventricular hypertrophy in females. Taken together, reversal of these deleterious offspring outcomes may reduce the cardiometabolic disease risk in BPH/5 offspring. Therefore, the developmental, pathological, and transgenerational effects of PE and obesity should be investigated separately by sex.

Obesity is a risk factor of PE possibly due to low‐grade systemic inflammation (Fruh, 2017). Tumor necrosis factor alpha (*TNFa*) is a proinflammatory factor produced by immune cells in adipose tissue (Fantuzzi, 2005; Weisberg et al., 2003). Macrophages in WAT of obese individuals produce higher levels of *TNFa* and interleukin 6 (*IL‐6*) than macrophages in lean individuals (Fantuzzi, 2005). *IL‐6* is also a proinflammatory factor produced by macrophages and adipocytes in adipose tissue (Fantuzzi, 2005; Weisberg et al., 2003). It is an inflammatory marker that is known to also increase in obese individuals compared to lean subjects (Fantuzzi, 2005). It has been estimated that WAT contributes to approximately 30% of circulating *IL‐6*, with visceral WAT producing higher levels of *IL‐6* compared with subcutaneous WAT (Fain et al., 2004). Prostaglandin‐endoperoxide synthase 2 (*Ptgs‐2*) encodes cyclooxygenase‐2 (*COX‐2*) and is a proinflammatory factor produced by macrophages in adipose tissue. Like *IL‐6* and *TNFa*, *Ptgs‐2* is also upregulated in WAT of obese subjects (Wang & Nie, 2016). In this study, BPH/5 AL/AL^F^ and AL/AL^M^ offspring have comparable subcutaneous WAT mass, that is attenuated in both sexes when born to pair‐fed dams. BPH/5 AL/AL^F^ offspring have excessive visceral reproductive WAT with elevated levels of *Ptgs‐2*, *TNFα*, and *IL‐6* that are attenuated when BPH/5 dams are pair‐fed during the first half of pregnancy. This suggests that further transcriptomic differences exist between sexes that may contribute to female obesity and early cardiovascular risk not observed in BPH/5 males.

Amarasekara et al. suggested that the maternal gut microbiome may contribute to the development of PE by exaggerating the inflammatory response (Amarasekara et al., 2015). Intriguingly, BPH/5 adult PF/AL^F^ and PF/AL^M^ offspring had lower mean arterial pressure and altered fecal microbial communities compared to ad libitum born offspring. The change in the fecal microbiome may be indicative of reprogrammed metabolism in BPH/5 offspring born to pair‐fed dams. These together may serve to improve signs of systemic cardiovascular disease, including heart enlargement and hypertension, and possibly even PE in subsequent generations of BPH/5. While the mechanisms are poorly understood, researchers believe the microbiome alters metabolism and modulates the weight gain in both the mother and the offspring potentially due to the in utero environment (Gohir et al., 2015).

Reprogramming BPH/5 male and female offspring through maternal weight loss may be done through several different mechanisms, including altered sex steroid hormone signaling, epi/genetics, and also sex‐specific modifications in placental nutrient transporters. Male and female sex and age‐specific differences in sex steroid hormones exist as they influence cardiovascular disease risk (Maggi & Della, 2018). For example, postmenopausal women with reduced estrogen and increased androgens are highly susceptible to hypertension (Barton & Meyer, 2009), whereas a drop in androgens is associated with age‐related hypertension in men (August & Oparil, 1999). Elevated androgens may promote oxidative stress and hypertension in both males and females (Reckelhoff & Roman, 2011). We know BPH/5 adult females have lower circulating 17β estradiol when in proestrus (Sutton et al., 2017), but androgens in both BPH/5 males and females have not been published. Furthermore, little is known regarding the genetics and epigenetics in BPH/5 mice. A recent genome wide study in BPH/5 females (Sones et al., 2021) revealed several genetic mutations shared between BPH/2 and BPH/5 that could contribute to the hypertensive phenotype, but functional studies are lacking. Finally, epigenetic modifications may exist on the X chromosome that may contribute to sex differences in BPH/5. Female offspring inherit two X chromosomes and inactivation of one is necessary to prevent overdose of X‐linked genes. Several cardiometabolic diseases have been associated with X overdose and sex‐biased gene expression (Arnold et al., 2008; Carrel & Willard, 2005; Reue, 2017). This may contribute to the BPH/5 offspring differences found in this study and are currently under investigation in the laboratory.

**FIGURE 6:**
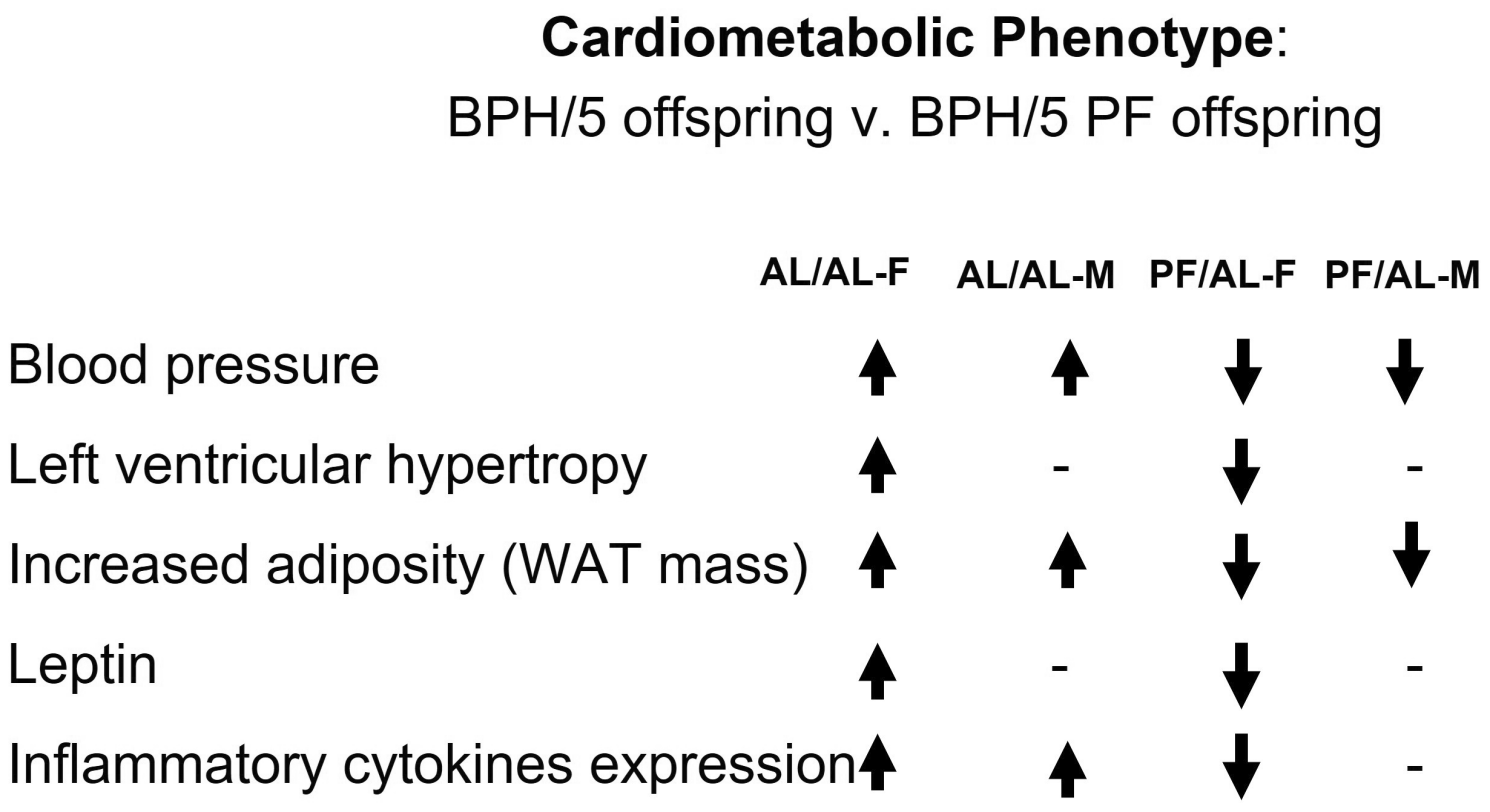
Summary of BPF/5 offspring cardiometabolic phenotypes after maternal food intake interventions. BPH/5 female offspring born to ad libitum fed dams: AL/AL^F^, BPH/5 male offspring born to ad libitum dams: AL/AL^M^, BPH/5 female offspring born to pair‐fed dams: PF/AL^F^, and BPH/5 male offspring born to pair‐fed dams: PF/AL^M^. Abbreviated to AL/AL‐F, AL/AL‐M, PF/AL‐F, and PF/AL‐M

In conclusion, the BPH5 mouse model is a mouse model to study the life cycle and offspring outcomes of PE by sex. BPH5 male and female offspring both demonstrate cardiometabolic risk, hypertension and heart enlargement, but have sex‐dependent differences in features of obesity (Figure 6). Overall, offspring born to pair‐fed dams have reduced cardiometabolic phenotype. Future studies are warranted to investigate the influence of the X chromosome on female inheritance and sex steroids hormones as they impact these sex‐dependent differences. Obesity coupled with PE contributes to the life cycle of obesity and cardiovascular risk observed in PE pregnancies. The different outcomes in BPH/5 may be due to in utero programming in an adverse environment.

AUTHOR CONTRIBUTIONS

K.F.B., J.L.S conceptualized & designed experiments, K.F.B., C.S., J.P.F., V.G., J.L.S collected data, K.F.B., C.S., D.A., C.C.L, G.C., J.L.S. analyzed results, K.F.B., J.L.S drafted figures, K.F.B., C.S., J.P.F., D.A., V.G., C.C.L, G.C., J.L.S. approved final manuscript.

## FUNDING INFORMATION

NIH (P20GM135002).

## Supporting information


Table S1

Figure S1
