# Supplementary material for: Sex‐specific effects of maternal weight loss on offspring cardiometabolic outcomes in the obese preeclamptic‐like mouse model, BPH/5
Source: Physiol Rep. 2022 Sep 6;10(17):e15444. doi: 10.14814/phy2.15444 (PMC9446412; doi:10.14814/phy2.15444)
Supplement: Supplementary file 1 — Table S1 [file PHY2-10-e15444-s001.docx]

| Supplementary Table 1. |  |  |  |  |  |
| --- | --- | --- | --- | --- | --- |
|  | AL/AL^F^ | AL/AL^M^ | PF/AL^F^ | PF/AL^M^ |  |
| Three-week-old Body Weight (g) | 8.32±0.39 | 9.32±0.31 | 9.65±0.75 | 9.9±0.62 |  |
| Adult Body Weight (g) | 24.16 ± 0.750 | 26.31±0.662 | 20.59±0.519* | 21.73±0.377 ^#^ |  |
| Visceral peri-renal WAT (mg) | 363.7±19.41 | 187.4±32.91^*^ | 56.42±7.30 ^*^ | 60.44 ±4.80^*#^ |  |
| Subcutaneous WAT (mg) | 393.0±85.12 | 301.7±31.66 | 125.5±13.12 ^*^ | 196.1±10.03^*#^ |  |
| Visceral reproductive WAT (mg) | 1366±206.5 | 452.9±70.96^*^ | 229.7±39.29^*^ | 243.9±19.57^*#^ |  |
| Heart Mass (mg) | 166.8±12.77 | 190.2±5.25 | 135.1±5.09^*^ | 145.1±6.95^#^ |  |
| Leptin (ng/ml) | 12.6±3.10 | 1.693±0.18^*^ | 5.51±0.75^*^ | 3.628±0.60^*^ |  |
|  | *p<0.05 different from AL/AL^F^ |  |  |  |  |
|  | #p<0.05 different from AL/AL^M^ |  |  |  |  |
|  |  |  |  |  |  |
