# Supplementary figures and images for: Sex‐specific effects of maternal weight loss on offspring cardiometabolic outcomes in the obese preeclamptic‐like mouse model, BPH/5
Source: Physiol Rep. 2022 Sep 6;10(17):e15444. doi: 10.14814/phy2.15444 (PMC9446412; doi:10.14814/phy2.15444)

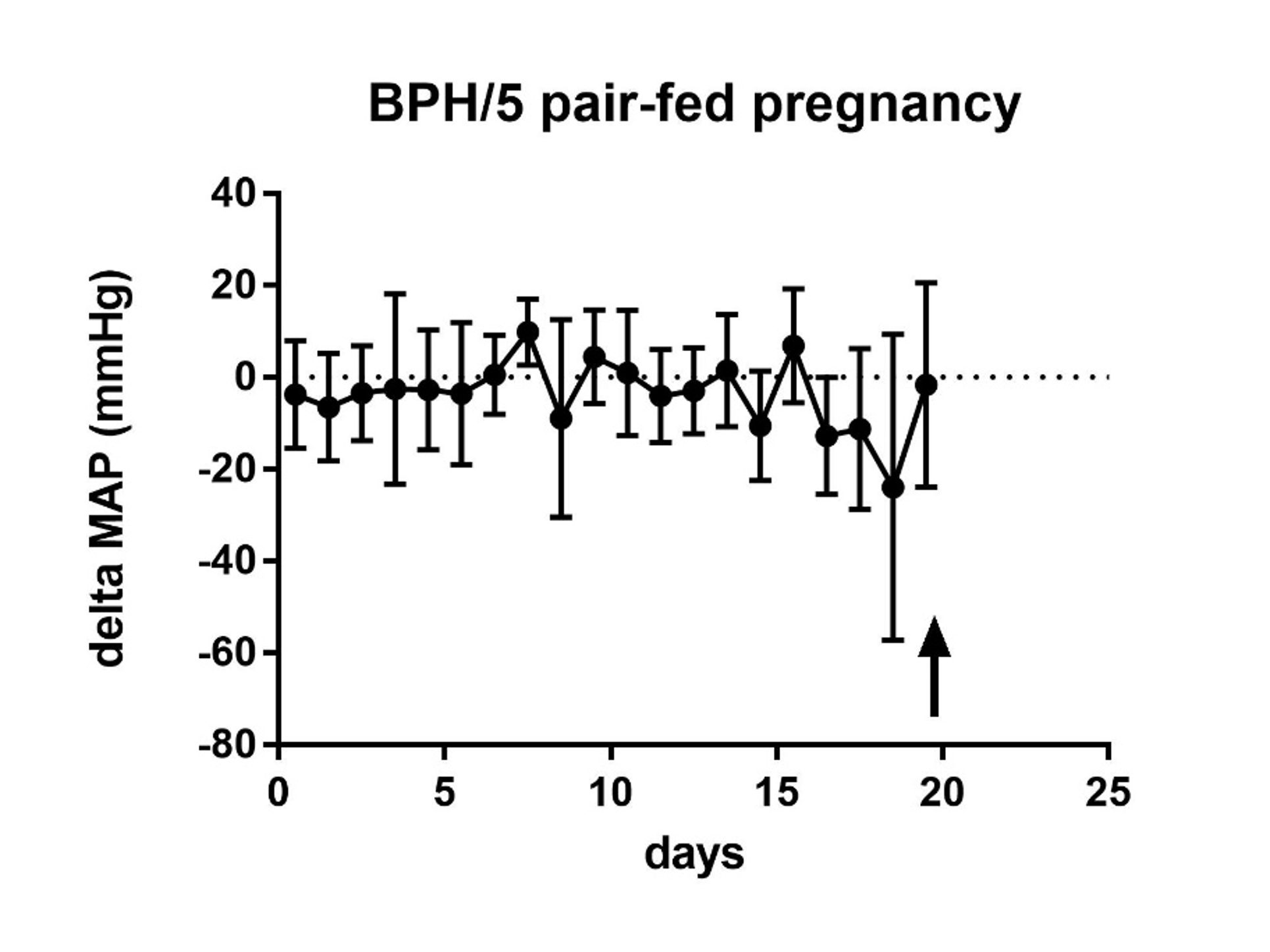

Supplement: Supplementary file 2 — Figure S1 [file PHY2-10-e15444-s002.tif]
